# The Surgery of the Intestines

**Published:** 1904-07-02

**Authors:** 


### THE SURGERY OF THE INTESTINES.

**Carcinoma of the Colon.**—Clogg[1] refers to the lymphatic involvment in these cases, and thinks that an attempt to remove the various groups of glands affected involves the sacrifice of more bowel than it is usually the custom to remove. With regard to this point he makes the following suggestions :—(1) In cæcal growths some inches of ileum with its mesentery should be removed. The greater part of the ascending colon should also be removed, practically to the hepatic flexure. (2) In growths in the region of the hepatic flexure he suggests the removal of the corresponding half of the transverse colon with as much of the mesentery as possible. (3) In the splenic region, including the growths just below and really in the upper part of the descending colon, the removal of the corresponding halves of the transverse colon and mesocolon should be practised. (4) In the recto sigmoid growths practically the whole of the meso-sigmoid should be removed, and this means a sacrifice of nearly the whole loop of bowel. In many, probably the majority of cases, this would entail the formation of an artificial anus, since it would be impossible to secure apposition of the cut ends of the bowel. Should this be so, the lower end of the bowel had better be sewn up. Walsham[2] gives reasons why he prefers to perform the operation for relief of carcinoma of the colon in two stages. The bowel above is distended and below it is contracted. If you perform the operation then you have a large bowel above to unite to a small bowel below, and that adds to the difficulty. The parts above, too, are inflamed and softened, and therefore are not in a good condition for holding the stitches, and the necessary opening of the bowel offers a chance of fouling the peritoneum. The general condition of the patient is also one that militates against such a prolonged operation as immediate resection and suture. Constipation has probably been marked, and there may have been vomiting for two or three days ; his tongue is furred, his breath is foul ; he is exhausted from lack of food, or, as is too often the case, he has been fed with nourishment injudiciously, and the food has been vomited, and his condition is as bad as it can be, for he is the subject of ptomaine poisoning or absorption. Another objection is, supposing you do not feel the carcinoma when you open the abdomen, you have to search for it, and to search among overcharged intestines is a serious matter, for a scratch of the gut in such circumstances may injure the peritoneal surface, and so allow the bacillus coli communis and other cocci which may exist in the fæces, and which have perhaps already invaded the mucous and submucous coats, to invade the peritoneum.

**Intestinal Obstruction.**—Lane[3] says that if a case of volvulus of the sigmoid is seen at a comparatively early date it may be quite unnecessary to excise the twisted loop. The reason of this is that the sigmoid, though considerably dilated, is not sufficiently inflamed, but that it can readily recover if material is able to escape freely from it. In such a condition we can but imitate the process of shortening up of the meso-sigmoid, which usually takes place to an extent sufficient to oppose the excessive elongation, dilatation of the loop, and the subsequent formation of a volvulus. To do this the loop is exposed and turned out of the abdomen and untwisted. An aperture is made into it if the material does not escape freely when it is untwisted and its contents are evacuated. The aperture in the gut is closed, and then its enormously hypertrophied outer longitudinal band of muscle-fibres is sutured to the edge of the oblique incision in the left groin, which, for this purpose, is made close to the anterior inferior spine of the ileum. Being fixed through a considerable portion of its length along its convexity, the sigmoid cannot again rotate and occlude its lumen. By adopting this method instead of excision we can avoid any such risk as is entailed by the removal of the distended loop. The same author[4] also speaks of an effectual means of dealing with the conditions of chronic obstruction of the large bowel resulting from the adhesions which develop in consequence of chronic constipation. Some of the cases were relieved by a thorough division of the bands and adhesions, and the complete liberation of the cæcum and ascending colon from the constricting influence exerted by them. In other cases where this treatment failed the ileum was connected with the sigmoid flexure by lateral anastomosis.

**Intestinal Perforation in Typhoid Fever** is fully treated of by Ashhurst,[5] who considers that although pain alone is not pathognomonic, it is by all means the most valuable subjective symptom of perforation. The pain is generally of a stabbing character, most frequently in the right lower quadrant of the abdomen; though for it to be felt in the epigastric or the umbilical regions is also customary, and a not unusual situation is in the bladder or, in the male, at the end of the penis. Davis[6] found the most marked symptoms were abdominal pain, localised tenderness, thoracic breathing, and muscular rigidity. Sudden decline of temperature and the phenomenon of shock had not been so constant. Rogers[7] says that the main point to be reiterated is that if one waits for a positive diagnosis, it will probably be too late to operate. In very few cases of typhoid fever can a diagnosis of perforation be made.

**Traumatic Rupture of the Intestines.**—Knaggs[8] points out the insidiousness of the symptoms produced by abdominal contusion when the bowel has been ruptured. The increasing rapidity of the pulse is undoubtedly a most important sign, but as it almost certainly indicates the absorption of toxins from the peritoneum, it means that valuable time has already been lost. A good deal of importance is attached to vomiting after abdominal contusion. It is true that even in cases of rupture of the bowel, it is not always present until unsatisfactory conditions are well advanced, but in doubtful cases, where a ruptured intestine is suspected, when the quiescent period following recovery from initial shock—if that has been present—is reached, the occurrence of vomiting, pointing to the probable implication of the peritoneum, may be the first sign upon which a fair amount of reliance can be placed. It may occur some time before the pulse rate has advanced sufficiently to make it quite clear that the increased frequency is not due to subsidiary causes. The presence of abdominal rigidity in these cases may appear to the surgeon to be easily explained by the contusion of the abdominal muscles.

[1] Practitioner, April, 1904, p. 525. [2] Lancet, Oct. 31, 1903, p. 1215. [3] Clin. Jour., Jan. 25, 1904, p. 213. [4] Lancet, Jan. 2, 1904, p. 19. [5] Ann. of Surg., Jan. 1904, p. 8. [6] Med. Rec., Jan. 23, 1904, p. 156. [7] Ibid. [8] Brit. Med. Jour., April 9, 1904, p. 832.

